# Regional alcohol consumption and alcohol-related mortality in Great Britain: novel insights using retail sales data

**DOI:** 10.1186/1471-2458-15-1

**Published:** 2015-01-07

**Authors:** Mark Robinson, Deborah Shipton, David Walsh, Bruce Whyte, Gerry McCartney

**Affiliations:** Public Health Science Directorate, NHS Health Scotland, 5 Cadogan Street, Glasgow, Scotland, UK; Glasgow Centre for Population Health, 94 Elmbank Street, Glasgow, Scotland, UK

**Keywords:** Alcohol consumption, Alcoholic beverages, Public health, Cross sectional studies

## Abstract

**Background:**

Regional differences in population levels of alcohol-related harm exist across Great Britain, but these are not entirely consistent with differences in population levels of alcohol consumption. This incongruence may be due to the use of self-report surveys to estimate consumption. Survey data are subject to various biases and typically produce consumption estimates much lower than those based on objective alcohol sales data. However, sales data have never been used to estimate regional consumption within Great Britain (GB). This ecological study uses alcohol retail sales data to provide novel insights into regional alcohol consumption in GB, and to explore the relationship between alcohol consumption and alcohol-related mortality.

**Methods:**

Alcohol sales estimates derived from electronic sales, delivery records and retail outlet sampling were obtained. The volume of pure alcohol sold was used to estimate per adult consumption, by market sector and drink type, across eleven GB regions in 2010–11. Alcohol-related mortality rates were calculated for the same regions and a cross-sectional correlation analysis between consumption and mortality was performed.

**Results:**

Per adult consumption in northern England was above the GB average and characterised by high beer sales. A high level of consumption in South West England was driven by on-trade sales of cider and spirits and off-trade wine sales. Scottish regions had substantially higher spirits sales than elsewhere in GB, particularly through the off-trade. London had the lowest per adult consumption, attributable to lower off-trade sales across most drink types. Alcohol-related mortality was generally higher in regions with higher per adult consumption. The relationship was weakened by the South West and Central Scotland regions, which had the highest consumption levels, but discordantly low and very high alcohol-related mortality rates, respectively.

**Conclusions:**

This study provides support for the ecological relationship between alcohol-related mortality and alcohol consumption. The synthesis of knowledge from a combination of sales, survey and mortality data, as well as primary research studies, is key to ensuring that regional alcohol consumption, and its relationship with alcohol-related harms, is better understood.

## Background

Per capita alcohol consumption levels are an important determinant of alcohol-related harm. For example, using time series analysis, a positive relationship between per capita consumption and liver cirrhosis mortality was observed within 13 of 14 countries for males in Western Europe [1]. However, there can be variation in rates of alcohol harm between populations with similar levels of consumption suggesting other context-specific factors (e.g. drinking cultures, drinking patterns) are also important [2].

Regional differences in levels of alcohol-related harm exist across Great Britain (GB) [3]. A consistent north–south divide has been observed over the past decade with alcohol-related harms highest in Scotland, North East and North West England [4]. The most obvious proximal driver of this geographical inequality is differences in levels and patterns of alcohol consumption. In England, such a relationship is partly supported by self-report survey data. Twigg and Moon used data from the Health Survey for England to show significantly higher rates of ‘binge drinking’ (defined as drinking eight units (64 g) of alcohol or more for men, and six units (48 g) of alcohol or more for women, on the heaviest drinking day in the past week) in England’s northern regions compared with the national average after adjustment for individual and area-based sociodemographic factors [5]. In contrast, binge drinking was lower in London, the South East and South West. Weekly alcohol consumption levels have also been shown to be higher in the north [6, 7].

The distinction between Scotland and other regions is less clear. Despite having substantially higher levels of alcohol-related mortality, self-reported consumption estimates in Scotland are comparable with other GB countries and regions [4, 8]. In addition, estimates of self-reported consumption in northern English cities have been shown to be comparable to similarly deprived Scottish urban areas, yet alcohol deaths were more than twice as high in the latter [9]. Part of the reason for this incongruence between self-reported alcohol consumption and harms is likely to be due to biases pertaining to sampling, response rates, social desirability and recall, which can often lead to substantial underestimations of consumption [10].

Alcohol sales data enable a more objective and accurate estimate of alcohol consumption [11]. However, estimating alcohol consumption using sales data is subject to its own biases and limitations. These include: retailer non-response; wastage and spillage; non-inclusion of some alcohol sales outlets; consumption by tourists; and unrecorded alcohol (e.g. homemade or informally produced alcohol (legal or illegal), smuggled alcohol, alcohol intended for industrial or medical uses, alcohol obtained through cross-border shopping). The overall impact of these biases is such that actual population levels of consumption are likely to be underestimated [12].

To date, objective sub-national estimates of alcohol consumption in GB have not been available. The aim of this paper was to present, for the first time, sales-based population consumption estimates for regions across the whole of Great Britain, and to compare these with levels of alcohol-related mortality.

## Methods

### Alcohol retail sales

We obtained data on alcohol retail sales for two full calendar years, 2010 and 2011, from market research specialists Nielsen and CGA Strategy (CGA) (hereafter ‘Nielsen/CGA’). Off-trade alcohol sales estimates (alcohol sold for consumption off the premises) were provided by Nielsen and produced from electronic sales records from most multiple retailers in GB (retailers with 10 or more retail shops operating under common ownership) and from a weighted stratified random sample of smaller ‘impulse’ retailers (retailers in which the consumer mainly uses the store for impulse or top-up purchases i.e. not the main grocery shop). Multiple retailers accounted for approximately three-quarters of total off-trade alcohol sales. On-trade alcohol sales data were obtained from market research specialists, CGA Strategy, whose estimates are based on a combination of delivery, sales, and survey data from a stratified sample of on-trade retailers. A more detailed description of the sampling methods used by Nielsen/CGA to collect alcohol sales data is provided elsewhere [13]. We have previously performed a detailed critique of the validity and reliability of alcohol retail sales data for monitoring population levels of alcohol consumption in Scotland [12]. The uncertainty in calendar year estimates of per adult sales is small and a lack of substantial year-on-year variability provides support for the precision of the estimates. This reflects the large proportion of alcohol sales data that are obtained using weekly store-census (electronic point-of-sales) data from the retailers who sell the most alcohol.

Mean alcohol sales in 2010 and 2011 were combined and analysed to minimise the impact of random fluctuations in annual estimates. The natural volume of alcohol sold (litres) was provided across seven drink categories: spirits, wine, beer, cider, perry, ready-to-drink beverages (RTDs), and fortified wine. We converted the volume of each drink category sold into pure alcohol volumes (litres of pure alcohol) using a category-specific percentage alcohol by volume (ABV). The ABV used was based on the typical strength of drinks sold in that category and was provided by the data suppliers. Per adult consumption was calculated by dividing pure alcohol volumes (litres of pure alcohol) by the total population aged 16 years and over [14, 15].

### Regional geographies

**Figure 1:**
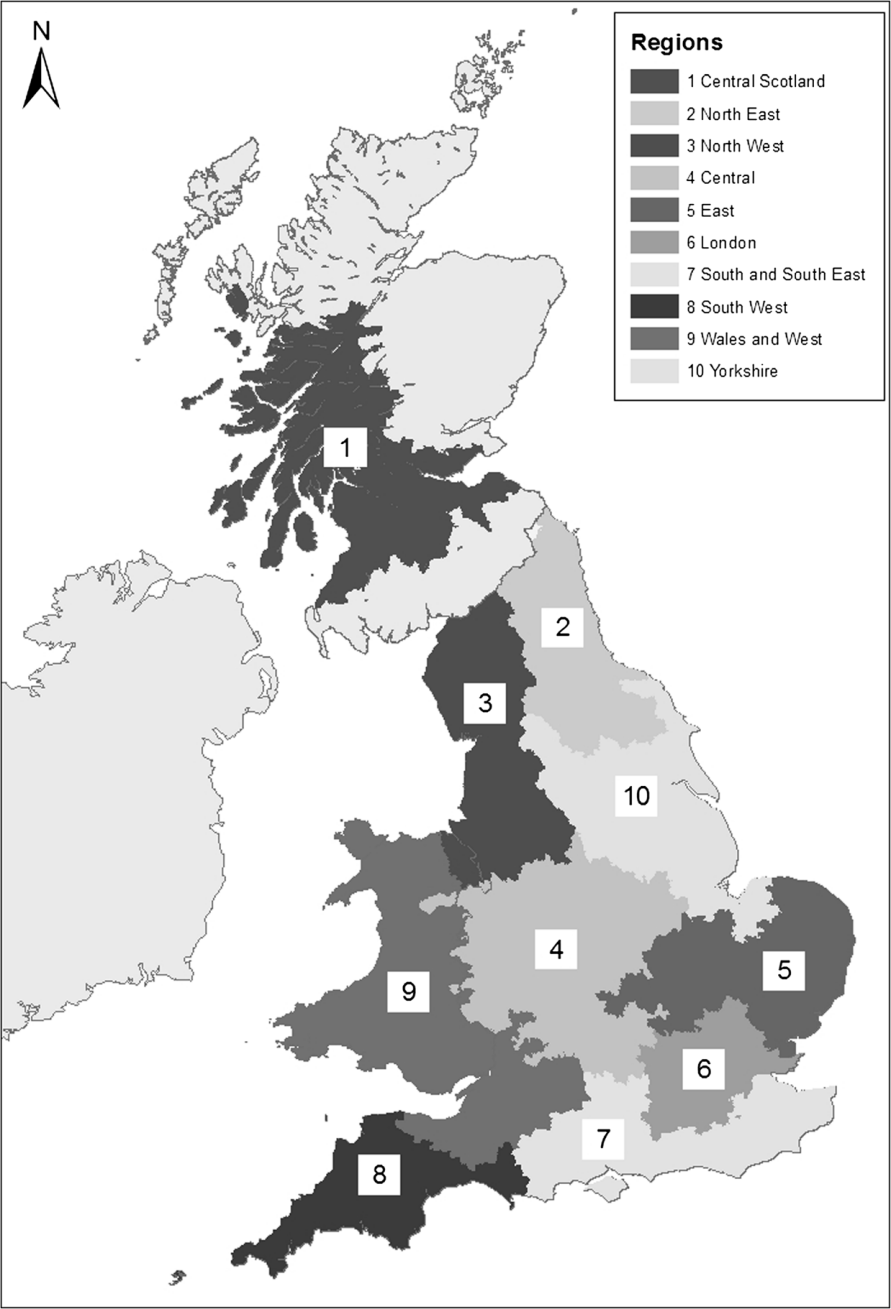
**Map showing regions included in this study** **(as defined by Nielsen/** **CGA).** This work is based on data provided through EDINA UKBORDERS with the support of the ESRC and JISC and uses boundary material which is copyright of the Crown.

Alcohol sales data were provided for Scotland and for England and Wales combined, as well as for the following regions (the regional population as a percentage of the total GB population is given in brackets): North West England (12%); North East England (5%); Yorkshire (10%); Central England (15%); East of England (7%); Wales and West of England (8%); London (21%); South West England (3%); South and South East England (10%); and Central Scotland (which includes most of urban Scotland (6%)) (Figure 1). Regions were defined by Nielsen/CGA according to postcode districts (e.g. G1, EH47, DH9). Alcohol sales data at smaller geographies were not available and regional boundaries could not be altered.

### Alcohol-related mortality and population data

Alcohol-related mortality was defined as those causes of death which are wholly attributable to alcohol using the standard Office for National Statistics (ONS) definition (Table 1) [3]. Data were directly age- and sex-standardised to the 1976 European standard population.

**Table 1:** **National statistics definition of alcohol**-**related deaths** (**ICD**-**10**)

ICD-10 code	Text
F10	Mental and behavioural disorders due to use of alcohol
G31.2	Degeneration of nervous system due to alcohol
G62.1	Alcoholic polyneuropathy
I42.6	Alcoholic cardiomyopathy
K29.2	Alcoholic gastritis
K70	Alcoholic liver disease
K73	Chronic hepatitis, not elsewhere classified
K74	Fibrosis and cirrhosis of liver (excluding K74.3-K74.5)
K86.0	Alcohol induced chronic pancreatitis
X45	Accidental poisoning by and exposure to alcohol
X65	Intentional self-poisoning by and exposure to alcohol
Y15	Poisoning by and exposure to alcohol, undetermined intent

For England and Wales, national and regional alcohol-related mortality data for 2010 and 2011 combined were provided by ONS. An output area to postcode district lookup file was used to aggregate population and mortality data to postcode district level. Data were then aggregated to a regional level based on the postcode districts included in the Nielsen/CGA definition. Population denominator data were drawn from the 2011 census [15].

For Scotland, alcohol-related mortality data and population estimates for 2010 and 2011 combined were available from National Records of Scotland [16]. Data were obtained at ‘data zone’ level (the smallest administrative geography for which required mortality and population denominator data were available, consisting of between 500 and 1,000 household residents) [17] to enable aggregation to postcode district and further to the Central Scotland region. The geographies were not coterminous meaning that a small number of data zones overlapped postcode districts that were on the boundary of Central Scotland. Analyses of population data showed that the net effect of these overlapping data zones was negligible and an acceptable ‘best-fit’ geography was achieved. A ‘Rest of Scotland’ geography was defined as those datazones not included in Central Scotland.

### Analysis

Alcohol retail sales data were available at aggregate level and so were analysed descriptively using tables and charts for the presentation of key comparisons. Per adult consumption was plotted against alcohol-related mortality and the Pearson correlation coefficient was determined to test the cross-sectional relation between the two variables. Sensitivity analyses explored the effect of omitting outliers from the correlation analysis.

## Results

**Figure 2:**
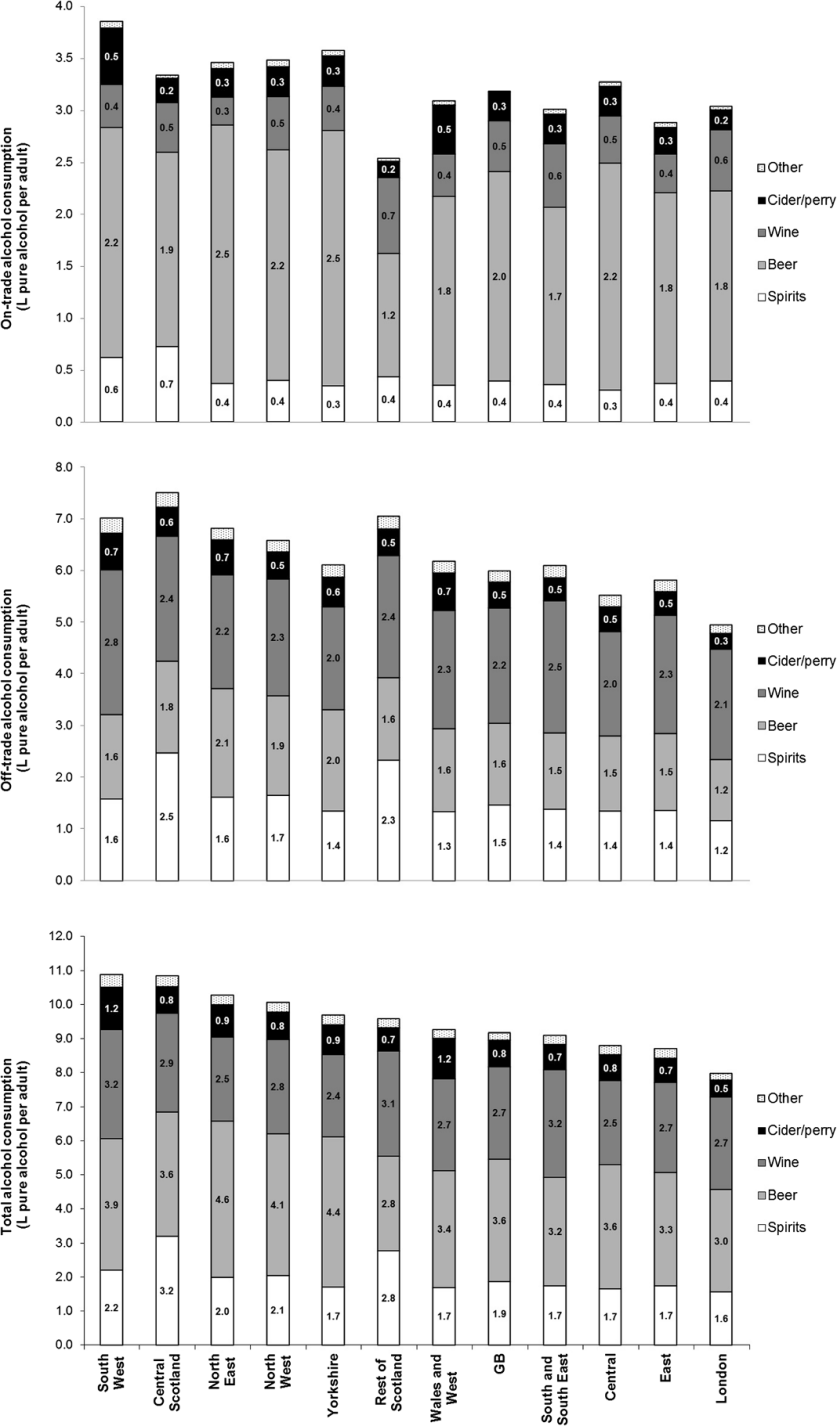
**Volume of pure alcohol sold per adult,** **by market sector,** **drink type and region,** **2010–** **11.** Other category consists of RTDs and fortified wine.

Of the 11 regions examined, the South West, Central Scotland, North East, North West and Yorkshire had notably higher per adult sales than the GB average (Figure 2). In contrast, there were lower per adult sales in London, Central England and the East of England. High per adult sales in the South West were driven by on-trade sales of spirits and cider, as well as off-trade wine sales. Both Scottish regions, but particularly Central Scotland, had substantially higher spirits sales than any other region in England and Wales, especially through the off-trade. In terms of market share, spirits sales accounted for 29% of the total market share in Scotland compared with ≤20% in the rest of GB. In Yorkshire and the northern English regions, beer was sold in higher volumes per adult than other GB regions, while cider sales were highest in the South West and Wales and West regions. London’s lower per adult sales were attributable to lower volumes of alcohol being sold in the off-trade. Compared with the GB average of 65%, the off-trade sector in London had marginally the lowest market share (62%); the highest off-trade market shares were in Scottish regions (69-74%).

**Figure 3:**
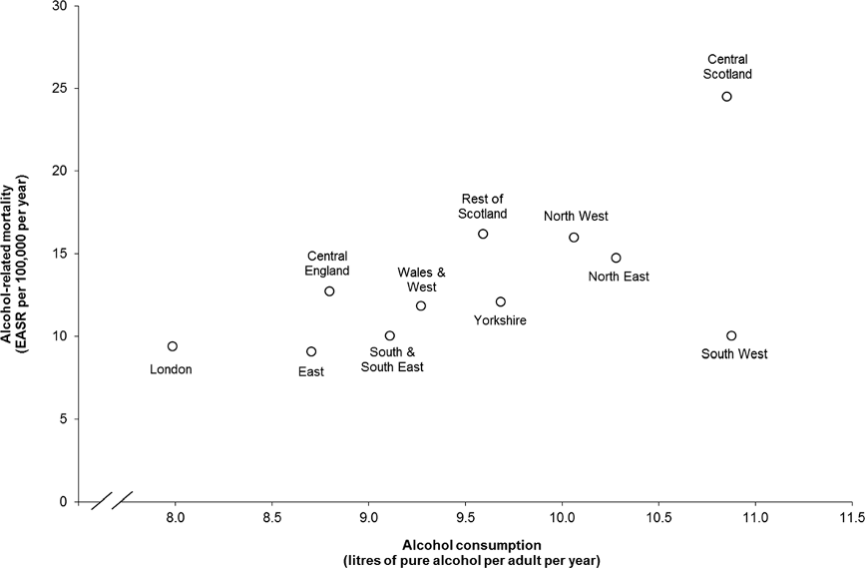
**Alcohol-** **related mortality** **(age/** **sex standardised)** **and per adult alcohol consumption,** **2010–** **11.**

Figure 3 compares regional levels of alcohol-related mortality and alcohol consumption. There was a general pattern of higher alcohol-related mortality in regions with higher population alcohol consumption (*r* =0.59, 95% confidence interval (CI) = -0.02 to 0.89). However, two opposing outliers were apparent. Despite similar levels of alcohol consumption in Central Scotland and South West England, alcohol-related mortality rates in Central Scotland were considerably higher and in South West considerably lower than might be expected based on the pattern across the other GB regions. If Central Scotland is removed from the analysis, the correlation weakens (r = 0.43, 95% CI = -0.27 to 0.84), while if the South West is omitted the correlation strengthens (r = 0.83, 95% CI = 0.43 to 0.96). The uncertainty around these coefficients is expectedly wide due to the small number of observations.

## Discussion

### Main findings

In this study, we have used alcohol sales data to present, for the first time, objective estimates of mean population consumption levels at sub-national geographies in Great Britain. We have confirmed the ecological relationship between consumption and harm; alcohol-related mortality rates are generally higher in regions with higher per adult consumption. However, atypical alcohol-related mortality levels in the South West and Central Scotland regions suggest regional-specific factors affect the consumption-harm relationship. There are some important differences in the types of alcohol sold through on- and off-licensed premises across GB regions. The high volume of alcohol sold per adult in the South West is driven by on-trade sales of cider and spirits and off-trade wine sales. In Scottish regions, a much higher volume of spirits is sold per adult than elsewhere in GB. Per adult sales in northern England are above the GB average and are characterised by high beer sales, while London has the lowest consumption, attributable to low off-trade sales across most drink types.

### Interpretation

It is well established that there is a positive relationship between average levels of alcohol consumption in a population and levels of alcohol-related harms [18]. However, the consumption-harm relationship is underexplored with the use of aggregate data within the UK. This is despite the fact that interventions aimed at reducing population levels of alcohol consumption, such as minimum unit pricing, have featured heavily in the policy landscape in recent years [19, 20]. Coghill *et al*. used data on the volume of alcohol cleared for sale to examine the association between per capita consumption and alcohol-related mortality [21]. Using UK level data for 1994–2008, the analysis provided evidence of a positive temporal association. While such time-series methods are generally more powerful than cross-sectional analyses, the study was limited by a relatively short time-series and the fact that the consumption data could not be disaggregated to lower levels of geography. In this study we have used aggregate data for a relatively small number of data points and shown a moderate relationship between consumption and mortality in spite of two apparent outliers. Thus, while the ecological analyses here are modest in scope and limit extensive interpretation, the findings are novel and provide a useful addition to the literature.

In 2007, the now defunct Association of Public Health Observatories published a detailed analysis of a wide range of alcohol-related indicators for Government Office Regions in England [6]. Using self-reported survey data, excessive alcohol consumption was shown to be consistently highest in the north, lowest in central and eastern regions, with regions in the south being around the middle. These patterns were found to concord with patterns in mortality and hospital admissions due to alcohol. Although the regions in our study are not directly coterminous with Government Office Regions, these patterns are broadly consistent with our results.

The key distinction between our findings and those in previous studies is alcohol consumption levels in Central Scotland and South West England. Use of the alcohol retail sales data has shown that per adult consumption in Central Scotland is higher than most other GB regions. Self-reported consumption estimates, either for Scotland as a whole or for subnational Scottish areas, are not noticeably different to other GB areas, despite alcohol-related mortality rates being substantially higher [4, 9]. In contrast, consumption estimates for the South West based on self-report data are usually about the same, or slightly lower than the national average [5, 6], which is consistent with what one would expect given the region’s profile of alcohol-related harms [6, 22]. However, in our study, we found the South West to have the highest level of per adult sales despite having one of the lowest rates of alcohol-related mortality. Detailed interrogation of potential reasons why different harm responses might exist for the same level of exposure is beyond the scope of this study. However, a few plausible explanations should be mentioned.

It is possible that the regional-level consumption estimates used in this study mask important differences in the distribution and patterns of alcohol consumption between regions. Aggregate sales data provide the most reliable source of overall consumption, but such estimates do not allow analyses of consumption levels and drinking patterns by different population subgroups (e.g. age, gender, social class, moderate/heavy drinkers) with different mortality risks. For example, it has been observed that despite no systematic differences in levels of alcohol consumption across socioeconomic groups, those of lower socioeconomic status experience much higher rates of adverse alcohol-related outcomes [23]. It is therefore possible that regions with higher levels of socioeconomic deprivation are more susceptible to alcohol-related harms than less deprived areas even if aggregate consumption levels are similar.

The high rate of deaths caused by alcohol in Central Scotland might have resulted from the drinking behaviours of a particular population cohort. For example, it has been hypothesised that political and economic changes in the 1980s had a particularly acute effect in Scotland (and particularly West Central Scotland) [24]. Specifically, rapid deindustrialisation and high levels of unemployment may have exposed a cohort of the population, particularly working-age men, to an increased risk of excessive alcohol use during this period. There was a sharp rise in alcohol-related mortality observed in Scotland in the 1990s, which was less evident in other GB regions [2]. Although the trend has been downward in recent years, the legacy of this earlier exposure might be partially responsible for the particularly high alcohol-related death rates relative to contemporaneous consumption levels in Central Scotland.

Regional differences in the type of alcohol consumed may also provide some important insights. The price of alcohol sold through the off-trade is much lower than through the on-trade [4]. Indeed, the increased affordability of off-trade alcohol since the 1980s has coincided with a change in purchasing patterns of consumers from on-trade to off-trade [4]. It is also known that heavier drinkers are more likely to consume cheaper alcohol [25]. In a recent study of patients with serious alcohol problems in Edinburgh, vodka (particularly cheap vodka) was found to account for the largest proportion of total consumption [26]. Previous analysis has shown that cheap spirits (particularly vodka) account for much of the additional volume of alcohol sold in Scotland compared with England and Wales [4].

In contrast to Scotland, the high per adult consumption in the South West was attributable to (more expensive) on-trade sales. These findings are consistent with a previous report which showed that the South West had the highest rate of on-licensed premises per 1000 population [6]. Off-trade wine sales were also highest in the South West and wine consumption is generally higher among those on higher incomes [27]. It is possible that these regional differences in beverage specific consumption represent differences in how and by whom alcohol is being consumed, which may impact on the risk of dying from an alcohol-related cause despite similar aggregate consumption levels. A more likely explanation is tourism. It is difficult to accurately quantify the impact of tourism on regional alcohol consumption estimates due to the availability of data for the bespoke geographies used in this study. Crude analyses of available data show that the South West region has the smallest resident population in our analyses, but has the highest rate of second addresses used for holidays by non-residents [28]. Furthermore, the South West has more incoming overseas tourists per head of the population than all other regions except London [29]. Thus, it seems reasonable to conclude that tourism would have more of an impact in the South West than most other regions. This provides another plausible explanation for its position as an outlier in the association between per adult consumption and alcohol-related mortality.

### Limitations

Alcohol sales estimates at smaller geographies would have been beneficial. The regions included in this study were large and included areas with very different health, social and deprivation profiles [30, 31]. Indeed, the Central Scotland region included 70% of the total Scottish population. Unfortunately, due to the sampling design used by the data providers, estimates at smaller geographies are not currently possible. This resulted in only a limited number of observations being available to explore the relation between consumption and harm within GB. Nonetheless, correlation analysis based on small numbers can be informative and, with appropriate caveats, should not preclude instructive interpretation and discussion [32]. In addition, as demonstrated by the likely tourism effect on the South West region in this study, data at smaller geographies can present other challenges.

Sales data over a longer time period would also have been useful to compare changes in consumption with changes in mortality. Future research that explores the temporal relation between consumption and harm at subnational geographies in the UK using time series analyses of aggregate data will enable more definitive conclusions to be drawn about causality.

We have assumed in this cross-sectional analysis that there is a contemporaneous association between levels of alcohol consumption and alcohol-related mortality. However, period and cohort effects (as alluded to earlier) as well as lag effects between changes in consumption and changes in harms could threaten this assumption. Nonetheless, an immediate change in alcohol-related mortality in response to fall in aggregate consumption is a consistent observation [33].

There are some important considerations when using alcohol-related mortality data, particularly at the subnational level. It is assumed that deaths occur in the same area in which individuals lived and consumed alcohol. This is not necessarily the case but the size of the regions analysed in this study is likely to have minimised the impact of this potential bias. There may also be variations in the attribution of death to alcohol across regions. However, a standard definition of alcohol-related deaths is used throughout GB, which should enhance inter-regional consistency. Furthermore, published estimates of alcohol-attributable deaths, which is a broader measure that also encompasses those conditions partially attributable to alcohol, results in a similar regional ranking as found in this study, although the geographies are not directly comparable (data not shown) [22].

## Conclusions

Overall, given the absence of evidence on aggregate consumption at subnational geographies in GB, and its association with related harms, this study provides a useful addition to the literature. We have provided evidence that within GB, alcohol-related mortality is generally higher in regions with higher levels of per adult consumption. From an alcohol research perspective, some consistent patterns between regional consumption estimates derived from self-report survey and sales data are reassuring. However, there are also some important distinctions. It is clear that no single source of alcohol consumption data is able to provide sufficiently representative and detailed data for monitoring and evaluation purposes. Rather, the synthesis of knowledge from a combination of routine sales, survey and mortality data, as well as primary research studies (both quantitative and qualitative), is key to ensuring that regional alcohol consumption and its relationship with alcohol-related harms, is better understood.

## Abbreviations

GB: Great Britain
ABV: Alcohol by Volume
ONS: Office for National Statistics.
